# Identification of a founder effect involving n.197C>T variant in *RMRP* gene associated to cartilage-hair hypoplasia syndrome in Brazilian patients

**DOI:** 10.1038/s41598-024-64407-8

**Published:** 2024-06-11

**Authors:** Maria Eduarda Gomes, Fernanda Kehdy, Fernanda Saloum de Neves-Manta, Dafne Dain Gandelman Horovitz, Maria Teresa Sanseverino, Gabriela Ferraz Leal, Têmis Maria Felix, Denise Pontes Cavalcanti, Juan Clinton Llerena, Sayonara Gonzalez

**Affiliations:** 1 Laboratório de Biologia Molecular/Medicina Genômica, Centro de Genética Médica Dr. José Carlos Cabral de Almeida & Serviço de Referência para Doenças Raras – Instituto Nacional de Saúde da Mulher, da Criança e do Adolescente Fernandes Figueira (IFF) – FIOCRUZ, Rio de Janeiro, Brazil; 2 Laboratório de Hanseníase da Fiocruz – Instituto Oswaldo Cruz (IOC) – FIOCRUZ, Rio de Janeiro, Brazil; 3 Unidade de Genética Clínica, Centro de Genética Médica Dr. José Carlos Cabral de Almeida & Serviço de Referência para Doenças Raras – Instituto Nacional de Saúde da Mulher, da Criança e do Adolescente Fernandes Figueira (IFF) – FIOCRUZ, Rio de Janeiro, Brazil; 4 Serviço de Genética Médica, Hospital de Clínicas de Porto Alegre (HCPA), Porto Alegre, Brazil; 5 Serviço de Genética Médica, Instituto de Medicina Integral Prof. Fernando Figueira, Recife, Brazil; 6 Grupo de Displasias Esqueléticas, Genética Médica, Departamento de Medicina Translacional, FCM – UNICAMP, Campinas, Brazil; 7 INAGEMP – Instituto Nacional de Genética Médica Populacional, Porto Alegre, Brazil; 8 Faculdade de Medicina Fundação Arthur Sá Earp Jr, Petrópolis, Brazil

**Keywords:** Disease genetics, Haplotypes, Rare variants

## Abstract

Cartilage-hair hypoplasia syndrome (CHH) is an autosomal recessive disorder frequently linked to n.72A>G (previously known as n.70A>G and n.71A>G), the most common *RMRP* variant worldwide. More than 130 pathogenic variants in this gene have already been described associated with CHH, and founder alterations were reported in the Finnish and Japanese populations. Our previous study in Brazilian CHH patients showed a high prevalence of n.197C>T variant (former n.195C>T and n.196C>T) when compared to other populations. The aim of this study was to investigate a possible founder effect of the n.197C>T variant in the *RMRP* gene in a series of CHH Brazilian patients. We have selected four TAG SNPs within chromosome 9 and genotyped the probands and their parents (23 patients previously described and nine novel). A common haplotype to the n.197C>T variant carriers was identified. Patients were also characterized for 46 autosomal Ancestry Informative Markers (AIMs). European ancestry was the most prevalent (58%), followed by African (24%) and Native American (18%). Our results strengthen the hypothesis of a founder effect for the n.197C>T variant in Brazil and indicate that this variant in the *RMRP* gene originated from a single event on chromosome 9 with a possible European origin.

## Introduction

Cartilage Hair Hypoplasia syndrome (CHH) is an autosomal recessive disease initially described by McKusick et al., 1965 in the Amish community^1^. Clinical signs include disproportionate short stature, hypotrichosis, anemia, impaired spermatogenesis, and Hirschsprung disease^2–5^. Patients often present immunological deficiencies with recurrent infections, higher incidence of autoimmunity diseases, and predisposition to cancer, in special lymphomas, reducing life expectancy^6–8^. Radiologically, it is observed short, splayed, and irregular metaphysis, especially at the knee regions. Although metaphyseal changes are the most expressive radiological feature, other radiological findings include short and thick tubular bones, short metacarpals and phalanges with cone-shaped epiphysis, curved femora, lumbar lordosis, and scoliosis^9^. Variations in the clinical phenotype inter-patients and even intra-families can be remarkable^10^. Also, CHH is one of thirty-six genetic diseases more prevalent in Finland (https://findis.org/)^11^ with an incidence estimated in 1/23,000 births in this population^12^. Prevalence for other geographical locations is not available.

CHH is characterized by biallelic pathogenic variants in the *RMRP* (mitochondrial RNA-processing endoribonuclease) gene located on chromosome 9p13^13^. This gene encodes a long non-coding RNA which is the core of RNAse MRP endoribonuclease^14^ responsible for many functions, such as rRNA processing, mitochondrial DNA replication and cell cycle progression^15–18^. Alterations can occur in the regulatory and transcribed regions^13^. Although CHH has been described more than 60 years ago, the genotype–phenotype correlation is still not well understood^7^. Currently, it is estimated that there are over 133 causative pathogenic variants in the *RMRP* gene^19^. Founder variants have already been described, such as n.72A>G variant, reported in the Finnish and Amish patients with CHH. This substitution accounts for 92% of the Finnish patients and it is present in 48% of non-Finnish Europeans patients either in homozygous or in compound heterozygous state^10^. Two other variants were associated with the Japanese population, n.220A>G and 17-bp dup at + 5^20^. In a previous report of our group^21^, we described a cohort of 23 Brazilian patients with CHH and several pathogenic variants. Furthermore, we observed an unexpectedly high prevalence of the n.197C>T variant, unlike that observed in patients of other nationalities^10,22–24^, suggesting a possible founder effect (FE) in this population. This work aimed to investigate the hypothesis of a common ancestral origin of the n.197C>T variant in Brazil.

## Results

### Detection of *RMRP* variants

The current cohort is composed of 32 Brazilian patients with CHH, from different regions of Brazil (Northeast, Southeast, South and Federal District); being 23 patients previously evaluated^21^ and nine novel patients recruited in the present study. All genotypes are described in Table S1 (supplementary material online). It is noteworthy that 70% of the cohort (21/30), considering only one propositus in familial cases, present the n.197C>T variant in compound heterozygosity. On the other hand, only one patient presented the n.72A>G variant associated with a duplication in the regulatory portion of the *RMRP* gene (previously described by Ridanpää et al.^10^). Furthermore, no patient with homozygous genotype was observed.

### Genetic ancestry of the cohort and Haplotype analysis

The average genomic contribution of each parental population (African, European and Native American) in the cohort was 24%, 58%, and 18%, respectively (Table 1, Supplementary Table S2 online). As the presence of n.197C>T variant was recurrent in patients from different regions of Brazil and rare in other countries, the hypothesis was raised that this alteration would have a single ancestral origin.

**Table 1:** Individual ancestry averages analyzed with 3 parental populations generated by Software Structure.

Population samples		AFR	EUR	AME	No. of individuals
Reference population	AFRICANS (AFR)	0.996	0.002	0.002	105
	EUROPEANS (EUR)	0.002	0.994	0.004	158
	AMERINDIANS (AME)	0.002	0.024	0.975	64
Brazilian cohort	ALL PATIENTS WITH CHH ANALYZED	0.240	0.585	0.175	21

Allelic and haplotypic frequencies of the TAG SNPs based on 1000 Genomes data suggested the existence of 10 different haplotypes in Africans and Europeans (Table 2). This analysis also revealed that the region delimited by TAG SNPs was discriminatory between ancestral populations. The major haplotype in Europe (C/C/G/C) was less frequent in Africa (0.356 X 0.075, respectively). Conversely, the major haplotype in Africa (C/C/A/C) was less frequent in Europe (0.393 X 0.005, respectively).

**Table 2:** Haplotype frequencies from selected TAG SNPs for European and African populations; and for individuals of this cohort.

rs10972552	rs7021463	rs1339374	rs1361338	Frequency in Europeans*	Frequency in Africans*	Number of observations in the cohort			Frequency in cohort
						Total	With n.197C>T	Without n.197C>T	
T	C	G	A	0.325	0.148	30	17	13	0.556
T	C	G	C	0.001	0.016	1	0	1	0.018
T	G	G	C	0.129	0.350	9	0	9	0.167
T	G	G	A	0.002	0.000	0	0	0	0.000
C	C	A	A	0.177	0.013	4	0	4	0.074
C	C	G	C	0.356	0.075	8	1	7	0.149
C	C	G	A	0.003	0.004	1	0	1	0.018
C	C	A	C	0.005	0.393	1	0	1	0.018
C	G	G	C	0.002	0.001	0	0	0	0.000
T	C	A	C	0.000	0.001	0	0	0	0.000
			Total	1.000	1.000	54	18	36	1.000

In gray are marked the most frequent haplotypes in the European and African population. *data from 1000 Genomes database (N = 504 EUR; N = 503 AFR).

**Figure 1:**
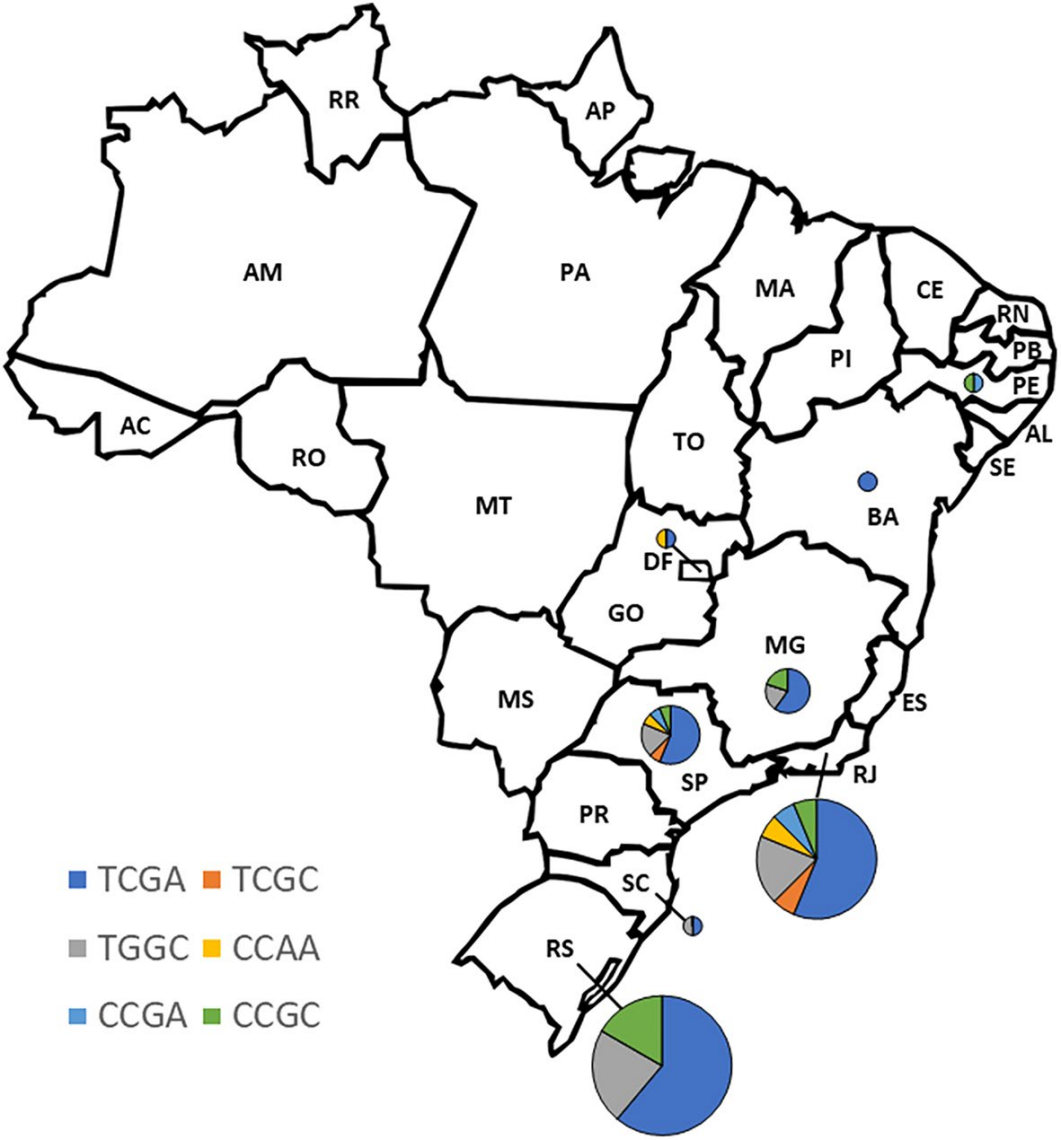
Geographic Distribution of Haplotypes in Brazil. This figure illustrates the frequency of observed haplotypes across different states in Brazil. Individual haplotypes of the patients were determined through Sanger sequencing of selected TAG SNPs. Following haplotype counting, they were categorized based on their geographical location within the country. The size of the pie charts is proportional to the quantity of haplotypes observed in each region.

Haplotype analysis was performed on samples from 27 unrelated patients from different regions of Brazil (Fig. 1), 18 being carriers of the n.197C>T variant (Table 2, supplementary Table S3 online). Siblings from the same family were expected to have the same haplotype; and, therefore, they were counted only once. Also, three patients carrying n.197C>T in compound heterozygosity were excluded due to insufficient material for haplotype analysis (patients 12, 13 and 28). The sequencing data of selected markers indicated a total of 7 distinct haplotypes in the Brazilian families. Of the 10 haplotypes inferred by Haploview, three were not found: T/G/G/A (frequency of 0.02% only in Europeans), C/G/G/C (frequency of 0.1% only in Africans), and T/C/A/C (frequency of 0.2% Europeans and 0.1% Africans).

Seventeen out of eighteen chromosomes with the n.197C>T variant presented the T/C/G/A haplotype (94.4%) in comparison to 13 of the 36 chromosomes carrying the remaining variants analyzed (36.1%).

Also, all chromosomes carrying the n.97_98dup2(TG) and n.-25_-4dup22(TACTACTCTGTGAAGCTGAGAA) segregated within T/C/G/A (n = 4) and T/G/G/C (n = 6) haplotypes, respectively. Interestingly, the only chromosome carrying n.72A>g variant in our cohort (patient 10) presented the C/C/A/G haplotype, the most frequent in individuals from Europe (Table 2).

The haplotype diversity of all analyzed chromosomes was 0.64 (Table 3). On the other hand, when specifically observing the group of chromosomes that contained the n.197C>T variant, this value was considerably lower (0.11), especially when compared to the group of haplotypes that did not harbor this variant (0.76). In addition, the diversity for parental populations for the constructed panel of markers (based on data from the 1000 Genomes database) was 0.72 in Europe and 0.70 in Africa (Table 3). The association of the T/C/G/A haplotype with the n.197C>T variant for the Brazilian population of patients with CHH was demonstrated by Fisher's exact test (p < 0.001).

**Table 3:** Haplotypic diversity calculated for haplotypes from patients and 1000G data.

Data	Haplotipic diversity	Number of haplotypes
All analyzed chromosomes	0.64	54
Chromosomes with n.197C>T variant	0.11	18
Chromosomes without n.197C>T variant	0.76	36
Europeans (1000G data)	0.72	504
Africans (1000G data)	0.70	503

## Discussion

This work focused on the study of a possible founder effect from a single country cohort outside Europe of patients with CHH. Previously, our group reported the clinical and molecular profile of 23 Brazilian patients with CHH showing an unexpectedly high prevalence of n.197C>T variant in the *RMRP* gene suggesting a possible founder effect^21^. The inclusion of nine novel patients in the present cohort, in which all but one individual presented the n.197C>T variant in one allele, corroborates with our previous results. It is noteworthy that there was no homozygous genotype for the n.197C>T variant. Despite the genetic diversity of the Brazilian population, we observed a significant prevalence of this specific genetic alteration, n.197C>T, in 70% of the analyzed patients, recruited from research centers in different regions of Brazil from 2016 to 2023. Although not all regions of Brazil are represented, the significant prevalence of the n.197C>T alteration in a mixed-race patient population reinforces the occurrence of the founder effect.

The n.197C>T variant was first reported in 2002^22^ and since then, other studies showed this variant in compound heterozygosity genotype for individuals of different nationalities, in a frequency that did not exceed 11.1%^10,23,24^. In gnomAD v4.1.0 database (https://gnomad.broadinstitute.org/)^25^, the frequency of n.197C>T is 0.00003464 (24/692,778 alleles), being twelve alleles from Latin Americans, two from African/African-Americans, nine from non-Finnish Europeans and one from another ethnic group, reinforcing the low occurrence of this variant outside Brazil. On the other hand, there are 68 registers of this variant in a total of 65,000 alleles (frequency = 0.001) in the database of Mendelics (a private Genomic Laboratory of molecular genetics diagnosis in Brazil). Despite being a database that contains a clinical and numerical bias, this information reinforces that the frequency of this variant is still higher than expected among Brazilians. In ABraOM (https://abraom.ib.usp.br/index.php), another Brazilian database, composed by elderly healthy population there is only one register among 2,342 alleles (frequency = 0.000427).

Herein, we sequenced a group of genetic markers flanking the *RMRP* gene to determine if the n.197C>T variant occurred on a shared haplotype among patients. Considering that such a set of markers were selected to be highly diversified, it was expected a similar variability between the population of this study and the ancestral populations (0.70 in Africans and 0.72 Europeans) for the delimited region for all chromosomes analyzed. However, a similar level of haplotypic diversity was observed only for chromosomes non harboring the n.197C>T (Hd = 0.76). Interestingly, those harboring the variant showed a dramatic reduction in haplotypic diversity (0.11), reinforcing the hypothesis of a common ancestry for n.197C>T.

Our findings reveal that the n.197C>T variant is present within two distinct haplotypes (T/C/G/A and C/C/G/C), separated by two mutational steps. These variations likely stem from the most prevalent haplotype, T/C/G/A. Notably, the emergence of the C/C/G/C haplotype appears to be a result of mutations originating from the dominant T/C/G/A haplotype. If this were not the case, we would anticipate encountering the n.197C>T variant associated with a broader array of haplotypes, suggesting independent mutational events. To further elucidate the evolutionary history of this variant, it would be insightful to analyze haplotypes from individuals outside of Brazil who carry the n.197C>T variant. Such analysis could help determine whether this variant arose from a single occurrence and subsequently spread globally. These assays could also help us to trace its origin and subsequent expansion in Brazil since this variant was identified in patients from all over the country without geographical constraints. However, conducting this type of study is time-consuming and involves significant challenges, including establishing international collaborations, navigating variable healthcare systems and genetic data availability, and managing differing regulations and ethical guidelines.

Clinical presentation of patients carrying the variant n.197C>T did not differ from patients with other combinations of genotypes in our cohort. Interestingly, no homozygotes carrying the variant n.197C>T were found; and, from a genetic counseling perspective an autosomal recessive inheritance risk of 25% of recurrence should always be considered. Clinical management for CHH syndrome patients carrying the n.197C>T variant should not differ from patients with other pathogenic *RMRP* genotypes; and should follow recommendations for surveillance of known complications, such as lymphomas; monitoring all children regardless of immune status during the first two years of life for recurrent infections, especially life-threatening varicella infection and for immune-deficiency risk factors^26^.

The n.197C>T alteration in the Brazilian patients probably occurs in an individual of European origin since T/C/G/A haplotype is the second most frequent in individuals from this continent (0.325 in European, Table 2). The Brazilian population has a genetic contribution of Africans, Amerindians, and Europeans and the means of genomic ancestry are 14.7%, 6.7%, and 78.5% respectively^27^. In accordance with the majority of studies in the Brazilian population, the inferences analysis showed that our patients present a predominantly European genetic contribution^28,29^. Nevertheless, the difference between European and Amerindian throughout the literature could be attributed to some bias in the panel of markers used^30,31^. Taken together, these data strongly suggest that n.197C>T variant probably derived from an isolated event and was transmitted from a common ancestor with a possible European origin.

Finally, other recurrent variants in our cohort also seem to be potential founder effects for the Brazilian population, such as the n.98_99dup2(TG) variant which segregates within the same haplotype (T/C/G/A) as the n.197C>T variant in all four patients who presented this duplication. Also, the n. -24_-3dup22(TACTACTCTGTGAAGCTGAGAA) is associated with the T/G/G/C haplotype (0.125 in Europeans and 0.350 in Africans). In both cases, the sample size was relatively small, which could lead to a selection bias. Therefore, more investigations with a larger number of individuals carrying these variants need to be conducted.

In conclusion, a total of 54 haplotypes were analyzed and the results revealed a major haplotype associated with n.197C>T variant related to CHH in Brazil. This strongly suggests the occurrence of a founder effect of this variant in the Brazilian population, which may even help the implementation of public health policies.

## Methods

### Ethics approval

This study was performed in line with the principles of the Declaration of Helsinki. Approval was approved by the Ethics Committee of Instituto Nacional de Saúde da Mulher, da Criança e do Adolescente Fernandes Figueira—IFF/Fiocruz (https://www.iff.fiocruz.br/index.php/atuacao/pesquisa?view=article&layout=edit&id=5), Hospital de Clínicas de Porto Alegre (https://www.hcpa.edu.br/pesquisa/comite-de-etica-em-pesquisa-cep) and Universidade Estadual de Campinas (https://www.fcm.unicamp.br/fcm/pesquisa/comite-de-etica-em-pesquisa/fale-conosco), under the number 1.557.698.

### Subjects and samples

Patients and their families were referred by their physicians from three Brazilian Medical Centers (Instituto Nacional de Saúde da Mulher, da Criança e do Adolescente Fernandes Figueira -IFF/Fiocruz; Grupo de Displasias Esqueléticas, FCM -UNICAMP and Hospital de Clínicas de Porto Alegre—HCPA).

Patients’ recruitment started in June 2016. A total of 32 patients and their family members were enrolled in this study. Twenty-three patients from our previous study^21^ and nine novel patients with positive results for CHH were included.

This is a retrospective and prospective study that was approved by the IFF/Fiocruz Ethical Committee Board under the number 1.557.698. The written informed consent for clinical and molecular analyses was obtained from all subjects.

Peripheral blood samples were collected from patients and their parents, when available. Genomic DNA was extracted by the salting-out protocol^32^.

### Admixture analysis

To infer the genomic ancestry of CHH Brazilian population we performed an individual admixture analysis using the AIMs (ancestry informative markers) panel. The inference of individual genomic percentages of African, European and Native American of 21 patients was performed by genotyping the 46-AIM-Indels multiplex according to the protocol described by Pereira et al.^30^. Fragments were detected using the ABI3500® sequencer (Applied Biosystems), and the generated products were analyzed using the GeneMapper ID v4.1 software. The individual ancestry estimates were calculated by STRUCTURE v2.3.4113 software, using HGDP-CEPH diversity panel as reference samples for ancestral populations^31^.

### Selection of TAG SNP markers

To determine whether all studied chromosomes carrying n.197C>T variant (rs948931144) shared the same haplotype, we analyzed a panel of markers distributed around the *RMRP* gene. For these, the SNPs present in the region spanning 7000 bp upstream and downstream of the *RMRP* gene were extracted from the 1000 Genomes database^33^. Their allelic frequencies in parental populations were calculated using PLINK software. Genomic annotation was performed by the ANNOVAR database. The SNPs were filtered based on the following criteria: frequency above 0.1 in Africans (Esan in Nigeria [ESN], Gambian in Western Division—Mandinka [GWD], Luhya in Webuye, Kenya [LWK], Mende in Sierra Leone [MSL], Yoruba in Ibadan, Nigeria [YRI] and Europeans (CEPH/Utah Collection [CEU], Finnish in Finland [FIN], British From England and Scotland [GBR], Iberian Populations in Spain [IBS], Toscani in Italia [TSI]); only SNPs located in the intergenic region, upstream, intronic or downstream region of the gene. The TAG-SNPs and the haplotype frequencies in these populations were identified by HAPLOVIEW (rs10972552, rs7021463, rs1339374 and rs1361338).

### Genotyping of TAG SNPs and data analysis

The region containing the TAG SNPs were PCR-amplified using patients and parental samples (PCR conditions available upon request) through Veriti 96-well thermal cycler (Thermo Fisher Scientific) and purified by High Pure PCR Product Purification Kit (Roche), according to the manufacturers’ instructions. Amplicons were sequenced by Sanger method on an automated DNA sequencer ABI 3730 (Applied Biosystems, Foster City, CA, USA) using BigDye v3.1 Sequencing Buffer (Applied Biosystems) as described by Otto et al., 2008^34^. Sequence data were analyzed using BioEdit Software version 7.2 (Ibis Biosciences, Carlsbad, CA, USA). For haplotype assembly, the gametic phase of each TAG SNP was inferred using sequence data from family pedigree. When parental samples were not available, allelic discrimination was determined in patients´ samples by cloning the entire region containing the selected markers into a pGEM-T Cloning Vector (Promega) before sequencing. Allele frequencies were calculated by direct counting and haplotype diversity was calculated (h = 1 − ∑p2), as proposed by Nei^35^. Fisher's exact test was used to compare the frequencies between haplotypes carrying or not the n.197C>T variant.

### Supplementary Information

Supplementary Information.

## Data Availability

The datasets generated during and/or analyzed during the current study are available from the corresponding author on reasonable request.
