# Supplementary material for: Identification of a founder effect involving n.197C>T variant in RMRP gene associated to cartilage-hair hypoplasia syndrome in Brazilian patients
Source: Sci Rep. 2024 Jun 11;14:13436. doi: 10.1038/s41598-024-64407-8 (PMC11166637; doi:10.1038/s41598-024-64407-8)
Supplement: Supplementary file 1 — Supplementary Information. [file 41598_2024_64407_MOESM1_ESM.pdf]

Identification of a founder effect involving n.197C>T variant in *RMRP* gene associated to Cartilage-Hair Hypoplasia syndrome in Brazilian patients

Maria Eduarda Gomes[1*], Fernanda Kehdy[2], Fernanda Saloum de Neves-Manta[2], Dafne Dain Gandelman Horovitz[3], Maria Teresa Sanseverino[4], Gabriela Ferraz Leal[5], Têmis Maria Felix[4], Denise Pontes Cavalcanti[6], Juan Clinton Llerena Jr.[3,7,8], Sayonara Gonzalez[1]

S1 Table: **Genotype of Brazilian patients with CHH (NCBI reference sequence: NR_003051.4)**

Sample ID	Allele 1	Allele 2
1	g.-20_-1dup20(ACTCTGTGAAGCTGAGGACG)	g.197C>T
2	n. -24_-3dup22(TACTACTCTGTGAAGCTGAGAA)	n.197C>T
3	n. -18_-2trip17(TCTGTGAAGCTGAGGAC)	n.195G>A
4	n.-23_-14dup10(ACTACTCTGT)	n.98_99dup2(TG)
5	n. -22_-13dup9(TACTCTGTG)	n.197C>T
6	n.98_99dup2(TG)	n.197C>T
7	n.11T>A	n.197C>T
**8**	**n.–21_-2dup20(TACTCTGTGAAGCTGAGGAC)**	**n.197C>T**
**9**	**n. -24_-3dup22(TACTACTCTGTGAAGCTGAGAA)**	**n.197C>T**
**10**	**n.-5_+5dup10(GGACGTGGTT)**	**n.72A>G**
**11**	**n.197C>T**	**n.246G>C**
**12**	**n.-18_-2dup17(TCTGTGAAGCTGAGGAC)**	**n.197C>T**
**13**	**n. -24_-3dup22(TACTACTCTGTGAAGCTGAGAA)**	**n.197C>T**
14	n.-18_-2dup17(TCTGTGAAGCTGAGGAC)	n. 182G>A
15	n.7G>T	n.197C>T
16#	n.37C>G	n.253A>C
17#	n.37C>G	n.253A>C
18	n. -12_+2dup14(AAGCTGAGGACGTG)	n.197C>T
19	n.-7_-6ins20(GGGACTACTCTGTGAAGCTG)	n.197C>T
20	n.-24_-3dup22(TACTACTCTGTGAAGCTGAGAA)	n.98_99dup2(TG)
21	n.98_99dup2(TG)	n.148G>C
22	n.7G>T	n.197C>T
23	n.-21_-2dup20(TACTCTGTGAAGCTGAGAAC) + n.262_332dup70	n.197C>T
24*	n. -24_-3dup22(TACTACTCTGTGAAGCTGAGAA)	n.197C>T
25*	n. -24_-3dup22(TACTACTCTGTGAAGCTGAGAA)	n.197C>T
26	n.37C>G	n. 99G>A
27	n. -24_-3dup22(TACTACTCTGTGAAGCTGAGAA)	n.197C>T
28	n.70_71ins1(T)	n.197C>T
**29**	n.–19_+4dup23(CTCTGTGAAGCTGAGAACGTGGT)	n.6C>T
**30**	**n.37C>T**	**n.197C>T**
**31**	**n. -24_-3dup22(TACTACTCTGTGAAGCTGAGAA)**	**n.197C>T**
**32**	**n.–23_-9dup15(ACTACTCTGTGAAGC)**	**n.197C>T**

Data previously published and available in Gomes *et al.,* 2019. New patients included in the cohort are indicated in bold. #,* indicate siblings;

S3 Table: Individual ancestry averages analyzed for 3 parental populations generated by Software Structure.

PATIENT	% AFR	% EUR	% AME
**1**	0.120	0.719	0.161
**2**	0.282	0.537	0.181
**3**	0.178	0.686	0.136
**4**	0.108	0.706	0.187
**5**	0.280	0.493	0.226
**6**	0.238	0.502	0.260
**7**	0.389	0.458	0.154
**14**	0.138	0.606	0.257
**15**	0.199	0.631	0.170
**16**	0.331	0.532	0.137
**17**	0.345	0.495	0.159
**18**	0.257	0.642	0.101
**19**	0.233	0.570	0.197
**20**	0.384	0.493	0.123
**21**	0.127	0.699	0.174
**22**	0.174	0.546	0.280
**23**	0.437	0.371	0.192
**24**	0.217	0.612	0.171
**25**	0.251	0.605	0.144
**26**	0.159	0.734	0.107
**27**	0.191	0.647	0.162

S3 Table: List of haplotypes found in patients

Patient ID	rs10972552	rs7021463	rs1339374	rs1361338	Variant
**1**	T	C	G	A	g.197C>T
	T	C	G	C	g.-20_-1dup20(ACTCTGTGAAGCTGAGGACG)
**2**	T	G	G	C	g. -24_-3dup22(TACTACTCTGTGAAGCTGAGAA)
	T	C	G	A	g.197C>T
**3**	T	C	G	A	g.195G>A
	C	C	G	A	g. -18_-2trip17(TCTGTGAAGCTGAGGAC)
**4**	C	C	G	C	g.-23_-14dup10(ACTACTCTGT)
	T	C	G	A	g.98_99dup2(TG)
**5**	T	C	G	A	g.197C>T
	T	G	G	C	g. -21_-13dup9(TACTCTGTG)
**6**	T	C	G	A	g.197C>T
	T	C	G	A	g.98_99dup2(TG)
**7**	C	C	A	A	g.11T>A
	T	C	G	A	g.197C>T
**8**	C	C	A	A	g.−21_-2dup20(TACTCTGTGAAGCTGAGGAC)
	T	C	G	A	g.197C>T
**9**	T	C	G	A	g.197C>T
	T	G	G	C	g. -24_-3dup22(TACTACTCTGTGAAGCTGAGAA)
**10**	C	C	A	A	g.-5_+5dup10(GGACGTGGTT)
	C	C	G	C	g.72A>G
**11**	T	C	G	A	g.197C>T
	C	C	G	C	g.246G>C
**14**	C	C	A	A	g.182G>A
	T	C	G	A	g.-18_-2dup17(TCTGTGAAGCTGAGGAC)
**15**	T	C	G	A	g.197C>T

	T	C	G	A	g.7G>T
**16**	C	C	G	C	g.37C>G
	C	C	A	C	g.253A>C
**18**	T	C	G	A	g.197C>T
	T	C	G	A	g. -12_+2dup14(AAGCTGAGGACGTG)
**19**	T	C	G	A	g.197C>T
	C	C	G	C	g.-7_-6ins20(GGGACTACTCTGTGAAGCTG)
**20**	T	C	G	A	g.98_99dup2(TG)
	T	G	G	C	g.-24_-3dup22(TACTACTCTGTGAAGCTGAGAA)
**21**	C	C	G	C	g.148G>C
	T	C	G	A	g.98_99dup2(TG)
**22**	T	C	G	A	g.197C>T
	T	C	G	A	g.7G>T
**23**	T	C	G	A	g.197C>T
	T	G	G	C	g.-21_-2dup20(TACTCTGTGAAGCTGAGAAC)
**25**	T	C	G	A	g.197C>T
	T	G	G	C	g.-24_-3dup22(TACTACTCTGTGAAGCTGAGAA)
**26**	T	C	G	A	g.99G>A
	T	C	G	A	g.37C>G
**27**	T	G	G	C	g.-24_-3dup22(TACTACTCTGTGAAGCTGAGAA)
	T	C	G	A	g.197C>T
**29**	C	C	G	C	g.37C>T
	C	C	G	C	g.197C>T
**30**	T	C	G	A	g.6C>T
	T	G	G	C	g.−19_+4dup23(CTCTGTGAAGCTGAGAACGTGGT)
**31**	T	C	G	A	g.197C>T
	T	G	G	C	g.-24_-3dup22(TACTACTCTGTGAAGCTGAGAA)
**32**	T	C	G	A	g.197C>T
	T	C	G	A	g.−23_-9dup15(ACTACTCTGTGAAGC)
